# Protocol for quantifying miRNA trafficking across the endosomal membrane

**DOI:** 10.1002/2211-5463.70315

**Published:** 2026-07-26

**Authors:** Syamantak Ghosh, Kamalika Mukherjee, Suvendra N. Bhattacharyya

**Affiliations:** ^1^ RNA Biology Research Laboratory, Molecular Genetics Division CSIR‐Indian Institute of Chemical Biology Kolkata India; ^2^ Department of Anesthesiology University of Nebraska Medical Center Omaha NE USA; ^3^ Department of Pharmacology and Experimental Neuroscience University of Nebraska Medical Center Omaha NE USA

**Keywords:** differential centrifugation, endosomes, *in vitro* miRNA loading assay, iodixanol density gradient, miRNA

## Abstract

The import of miRNA across the endosomal membrane is an essential prerequisite for the loading of miRNAs into the endosomal lumen. These miRNAs are then incorporated into mature multivesicular bodies (MVBs), which then fuse with the plasma membrane, leading to the release of exosomes or extracellular vesicles carrying miRNAs into the intercellular space. Here, we present a reproducible *in vitro* assay for the quantification of miRNA import across the endosomal membrane. We detail the isolation of intact endosomes from mammalian cells, followed by the loading of synthetic, chemically modified microRNAs (miRNAs) into the vesicles in an RNase‐resistant form. Integrating differential centrifugation and iodixanol density‐gradient fractionation, we isolate endosomes free of endoplasmic reticulum contamination. Then, we use optimized *in vitro* cargo‐loading techniques to facilitate subsequent assessment of miRNA uptake, stability, and delivery efficiency in the presence of altered external factors, such as ATP or substrate concentration. Ultimately, this protocol presents a standardized method for quantifying miRNA trafficking across the endosomal membrane.

## Graphical abstract

An *in vitro* protocol measures miRNA uptake into endosomes isolated from mammalian cell extracts, which are free of subcellular contaminants. Performed at 37 °C in the presence of ATP, it ensures the import of single‐stranded miRNA into the endosomal lumen. The imported miRNA is then recovered, quantified via qRT‐PCR after RNase treatment and re‐isolation of the endosomes to eliminate non‐imported material. Image initially generated with the FigureLabs Online platform, vectorized and modified for correctness.

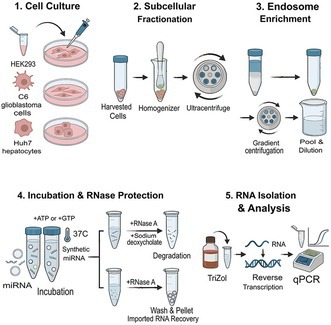

**Abbreviations**
AFM: atomic force microscopy
ALIX: ALG‐2 interacting protein X
ATP: adenosine triphosphate
BSA: bovine serum albumin
Cas9: CRISPR‐associated protein 9
CRISPR: clustered regularly interspaced short palindromic repeats
DMEM: Dulbecco's modified Eagle Medium
DTT: dithiothreitol
EDTA: ethylenediaminetetraacetic acid
EEA1: early endosome antigen 1
EGTA: ethylene glycol tetraacetic acid
ER: endoplasmic reticulum
EV: extracellular vesicles
Facl4: acyl‐CoA synthetase long‐chain family member 4
FBS: fetal bovine serum
HDL: high density lipoprotein
HEPES: 4‐(2‐hydroxyethyl)‐1‐piperazineethanesulfonic acid
HRS: hepatocyte growth factor‐regulated tyrosine kinase substrate
HuR: human antigen R
miRNA: microRNA
MVB: multivesicular bodies
NTA: nanoparticle tracking analysis
PBS: phosphate‐buffered saline
PMSF: phenylmethanesulfonyl fluoride
PNK: polynucleotide kinase
qRt‐PCR: quantitative reverse transcription polymerase chain reaction
Rab5: Ras‐related protein Rab‐5A
TBS: TRIS‐buffered saline
TCL: total cell lysate

miRNAs are important regulators of gene expression that can be packaged into extracellular vesicles (EVs) released by metazoan cells, thereby carrying specific miRNAs and facilitating the exchange of miRNA cargoes between cells [1]. The investigation of miRNA export via EVs requires rigorous controls to verify authenticity [1, 2, 3, 4]. To investigate extracellular miRNA export, it is crucial to adhere to specific experimental and technical standards. This ensures that the extracellular miRNAs detected result from an active, regulated export process rather than passive leakage from cell damage. It is important to identify and validate miRNAs that are actively exported by a cell line, a process that may involve packaging multiple miRNA cargoes into EVs [5, 6, 7, 8]. Additionally, demonstrating that miRNAs are actively secreted and enclosed within EVs or protein complexes, such as Ago2 or HDL—rather than passively released—is essential. Employing standardized protocols for EV and miRNA isolation and quantification, with normalization to appropriate endogenous controls, is also necessary [1, 9, 10, 11, 12]. Potential mechanisms to explore include investigating the roles of the Endosomal Sorting Complex Required for Transport (ESCRT) and proteins, such as Ago2 and GW182, in the selective packaging of miRNAs [10, 13]. Additionally, identifying specific nucleotide motifs that interact with RNA‐binding proteins to facilitate active sorting could provide valuable insights. Some studies also suggest that miRNAs involved in translational repression are selected for export in a manner that depends on their target RNAs [9, 14, 15, 16, 17, 18, 19, 20, 21].

The specific assay system requirement for studying the mechanism of export is evident, and the lack of a reproducible assay to monitor miRNA trafficking across the endosomal membrane poses a significant challenge. The previous assay system, which used membrane fractions from a cell lysate, is a very crude method for investigating this process and is susceptible to contamination by other membranous structures, such as the endoplasmic reticulum (ER), which can significantly contribute to noise in post‐assay miRNA quantification due to aberrant miRNAs associated with ER membranes [12, 22, 23]. Similarly, assays using artificial liposome‐like entities do not accurately reflect the physiological context required to study miRNA trafficking across membranous structures.

These apparent limitations must be addressed in the study of miRNA import into the endosomal lumen, a preliminary step before endosomes mature into large multivesicular structures that ultimately fuse with the plasma membrane, facilitating the release of exosomes or EVs containing miRNA cargoes. This was associated with changes that freed endosomes from contamination by mitochondria and the ER, thereby ruling out two important sources of miRNAs in mammalian cells. This starting material for the assay should be flexible enough to accommodate membrane‐associated proteins that must be recovered with endosomes, intact endosomes after the import assay, and easy recovery and quantification of the assay substrate RNA after the import reaction.

Utilizing differential Iodixanol gradient centrifugation to isolate endosome‐enriched fractions, free from mitochondrial and ER contamination, followed by an assay conducted in ATP‐containing buffer with a synthetic miRNA substrate, and subsequent RNase protection assay to recover and quantify the import‐protected RNA demonstrated a time‐ and ATP‐dependent import of miRNA into endosomes *in vitro*. During the endosomal import process, the endosomes also matured into larger MVB‐like structures, as evidenced by nanoparticle tracking analysis (NTA) and atomic force microscopy (AFM) studies conducted before and after substrate analysis.

## Materials

### Cell culture, cell homogenization, and isolation of endosomes

1. Dulbecco's modified Eagle's medium; Gibco, Life Technologies, Carlsbad, CA, USA (Cat no.: 12800‐017).
2. Heat‐inactivated fetal bovine serum (FBS); Gibco (Cat no.: 10082‐147).
3. Penicillin–Streptomycin; Gibco (Cat no.: 15140122).
4. Trypsin–EDTA; Gibco (Cat no.: 25200‐072).
5. 1× phosphate‐buffered saline (PBS) (pH = 7.4); Gibco (Cat no.: 10010‐023).
6. C6 (Rat Glial cell line); ATCC (Cat no.: CCL‐107).
7. HEK‐293; ATCC (Cat no.: CRL‐1573).
8. Microprocessor‐controlled air system class II biohazard safety cabinet; ESCO Make.
9. CO_2_ incubator; Innova Make.

### Cell lysate preparation and isolation of endosomes

1. OptiPrep™ Density Gradient Medium; Sigma‐Aldrich, St. Louis, MO, USA (Cat no.: D1556).
2. Sucrose Sigma S0389; Sigma‐Aldrich (Cat no.: S0389).
3. EGTA; Sigma‐Aldrich (Cat no.: E8145).
4. HEPES; Merck, Rahway, NJ, USA (Cat no.: H3537).
5. DTT; Roche, Mannheim, Germany (Cat no.: 10197777001).
6. Dounce Tissue Grinder, 2 mL; KIMBLE® KONTES® (Cat no.: 885300‐0002).
7. Ultracentrifuge; Beckman Coulter Life Sciences, Indianapolis, Indiana, USA (Optima L‐90K).
8. SW 60 Ti Swinging‐Bucket Rotor Package; Beckman Coulter Life Sciences (Cat no.: 335650).
9. 4 mL Open‐Top Thinwall Polypropylene Tube; Beckman Coulter Life Sciences (Cat no.: 328874).
10. 1.5 mL Open‐Top Thinwall Polypropylene Konical Tube; Beckman Coulter Life Sciences (Cat no.: 358117).
11. Refrigerated micro centrifuge with 1.5/2‐mL tube rotor; Eppendorf, New Castle, Delaware, USA.
12. Thermomixer Comfort with 1.5‐mL exchangeable thermoblock; Eppendorf.
13. Homogenization buffer: 0.25 m sucrose, 78 mm KCl, 4 mm MgCl_2_, 8.4 mm CaCl_2_, 10 mm EGTA, 50 mm HEPES (pH 7.0) supplemented with 100 μg·mL^−1^ of cycloheximide, 0.5 mm DTT, and 1× PMSF.
14. Optiprep/Iodixanol gradient solutions: 5, 10, and 15% Iodixanol solutions were prepared from 60% Optiprep solution in a buffer consisting of 78 mm KCl, 4 mm MgCl_2_, 8.4 mm CaCl_2_, 10 mm EGTA, and 50 mm HEPES (pH 7.0).

### RNA import assay and RNase protection

1. Synthetic single‐stranded RNA; Integrated DNA Technologies, Coralville, Iowa, USA:

miR‐122‐5p: 5′‐UGGAGUGUGACAAUGGUGUUUG‐3′
miR‐146a‐5p: 5′‐UGAGAACUGAAUUCCAUGGGUU‐3′

2 miRNA loading assay buffer: 0.25 M sucrose, 78 mm KCl, 4 mm MgCl_2_, 8.4 mm CaCl_2_, 10 mm EGTA, and 50 mm HEPES (pH 7.0) supplemented with 100 μg·mL^−1^ of cycloheximide, 0.5 mm DTT, and 1× PMSF.
3 Wash/Diluent buffer: 78 mm KCl, 4 mm MgCl_2_, 8.4 mm CaCl_2_, 10 mm EGTA, 50 mm HEPES (pH 7.0).
4 RNase A, DNase, and protease‐free; Thermo Scientific, Waltham, MA, USA (Cat no.: EN0531).

### RNA isolation and estimation

1. TRIzol Reagent; Thermo Fisher Scientific (Cat no.: 15596026).
2. TRIzol LS Reagent; Thermo Fisher Scientific (Cat no.: 10296010).
3. miRVANA miRNA Isolation Kit; Thermo Fisher Scientific, Waltham, MA, USA (Cat No. AM1560).
4. TaqMan Universal PCR Master Mix No AmpErase; Applied Biosystems (Cat No. 4324018).
5. TaqMan reagents for the target miRNA amplification; Applied Biosystems (Waltham, MA, USA), with assay IDs: miR‐122 (000445); miR‐146a (000468); miR‐155 (002571); let‐7a (000377); miR‐21 (000397); and U6 SnRNA (001973).

### Western blot analysis

1. Antibodies to endosome, late endosomes, and ER marker proteins: EEA1; Cell Signaling Technology (CST), Danvers, MA, USA, Cat no.: 2411S; Rab5, CST, Cat no.: 3547S; Alix, Santa Cruz Biotechnology, Dallas, TX, USA, Cat no.: sc53538; Calnexin, Bethyl Laboratories Inc. (BLI) Boston, MA, USA, Cat no.: A303‐696A; HRS, BLI, Cat no.: A300‐989A; Rab7a, CST, cat no.: 9367S; Facl4, Norvus Biologicals, Centennial, CO, USA, Cat no.: NBP2‐16401.
2. Secondary antibody: Horseradish peroxidase–conjugated secondary antibodies (1:8000 dilutions).
3. Western image development with West Pico Chemiluminescent substrate, Thermo Fisher Scientific, Cat no. 34577.
4. UVP BioImager 600 system coupled with VisionWorks Life Science software, version 6.8 (UVP).

## Methods

**Fig. 1:**
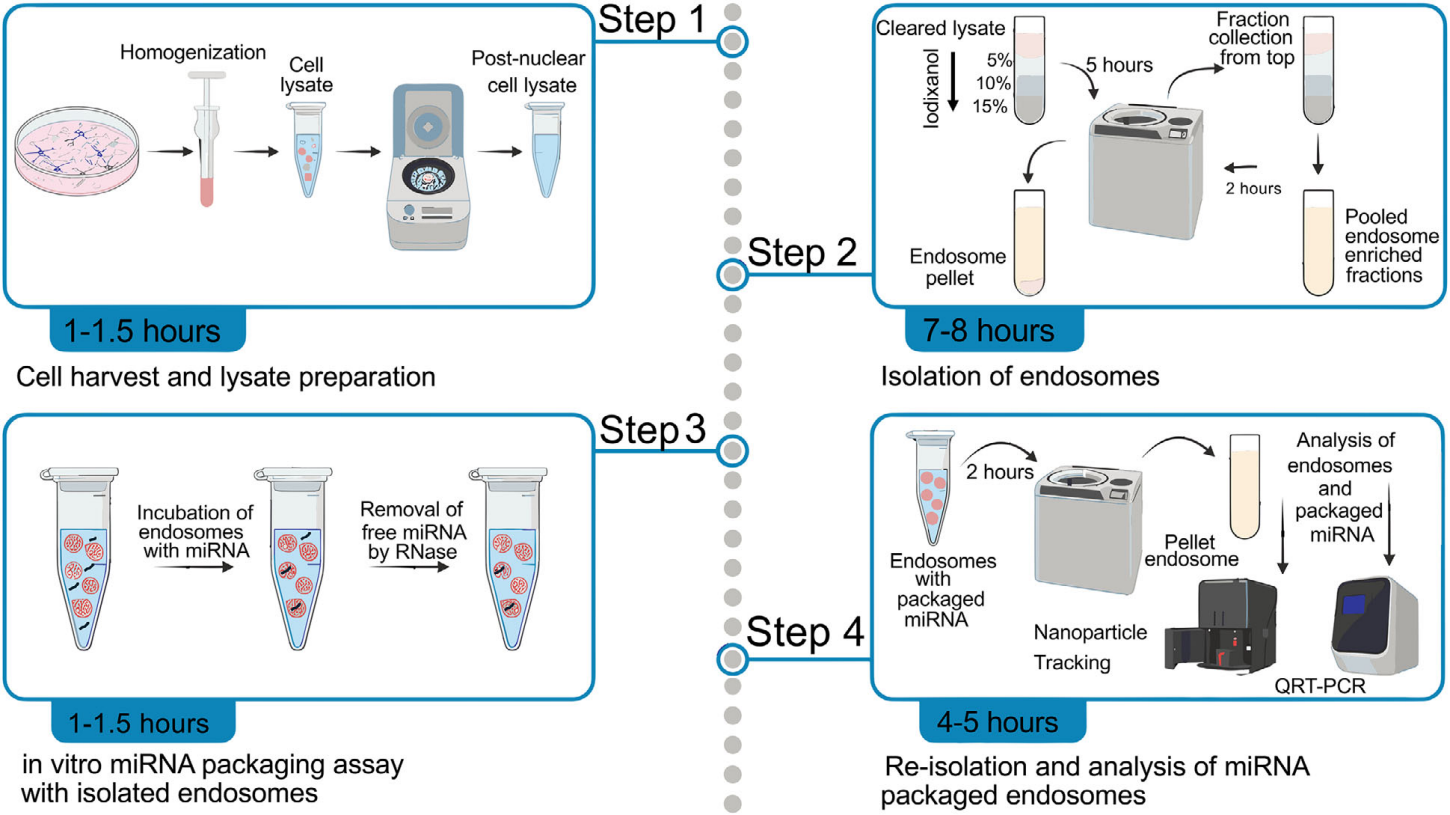
Schematic of the isolation of endosomes from C6 glioblastoma cells followed by an *in vitro* RNA import assay with synthetic microRNA (miRNA) as substrate. After incubation with substrate RNA, RNase treatment, and recovery of endosomes containing imported and RNase‐protected RNAs inside, the miRNAs were quantified by quantitative reverse transcription polymerase chain reaction (qRT‐PCR). The Sketch‐to‐Figure Feature of the FigureLabs online software was used to generate the initial image.

The isolation of endosomes, their maturation, and the loading of miRNAs in the *in vitro* reaction, followed by the recovery of endosomes after RNase protection of the imported RNAs and quantification in a time‐ and concentration‐dependent manner, constitute the overall strategy for developing this assay. A graphical summary (Fig. 1) of the assay development and data validation is presented, and detailed descriptions of each step have been provided in the subsequent sub‐sections.

### Isolation and characterization of endosomes

#### Stock & Buffer Formulations

1. Preparation of Buffers and solution. All buffers are to be prepared using autoclaved ultrapure water (≥18.2 MΩ·cm), filtered through a 0.22 μm vacuum filter, and stored at 4 °C unless otherwise noted.

- Complete DMEM medium: DMEM high glucose + 10% FBS + 1% Penicillin–Streptomycin (10 000 U·mL^−1^).
- 1× PBS (Harvest/Wash): Sterile, calcium/magnesium‐free PBS. To be stored at 4 °C.
- 10× Homogenization Stock Buffer: 500 mm HEPES, 780 mm KCl, 40 mm MgCl_2_, 84 mm CaCl_2_, and 100 mm EGTA. To adjust the pH to exactly 7.0 at room temperature using KOH or HCl.
- Working 1× Homogenization Buffer (Prepare fresh on ice daily):
  ○ 10× Stock to be diluted 1:10 in sterile ultrapure water.
  ○ Sucrose to be added to a final concentration of 0.25 m.
  ○ Crucial additives (Add immediately before use):
    • Cycloheximide (final concentration: 100 μg·mL^−1^)
    • DTT (final concentration: 0.5 mm)
    • PMSF (final concentration: 1× from stock [100 nm])
- Discontinuous Iodixanol (OptiPrep) Gradient Solutions: have to use OptiPrep (60% w/v Iodixanol stock) and dilute it with Working 1× Homogenization Buffer to generate the working percentages:
  ○ 15% Iodixanol: 1 part 60% OptiPrep + 3 parts 1× Homogenization Buffer.
  ○ 10% Iodixanol: 1 part 60% OptiPrep + 5 parts 1× Homogenization Buffer.
  ○ 5% Iodixanol: 1 part 60% OptiPrep + 11 parts 1× Homogenization Buffer.
- Endosome wash buffer: 50 mm HEPES (pH 7.0), 78 mm KCl, 4 mm MgCl_2_, 8.4 mm CaCl_2_, and 10 mm EGTA (Contains no sucrose or protease inhibitors).

2 Physical setup checklist.

- The ultracentrifuge and swing‐bucket rotor (e.g., SW41 Ti or equivalent) are to be prechilled to 4 °C.
- A low‐speed benchtop centrifuge to be prechilled at 4 °C.
- The 2‐mL glass Dounce homogenizer (mortar and tight‐fitting pestle Type A) is to be cooled in an ice‐water bath.
- An aliquot of 1× PBS and the freshly supplemented Homogenization Buffer is to be cooled on ice.

#### Part 1: Cell culture, scale‐up, and preparation

*Summary*: [Thaw/Flask Setup] → [Change Media Every 2 Days] → [Split @ 80–85% Confluency] → [Pre‐Assay Target: 80–85%]

1. Thawing & maintenance: Thaw a vial of HEK293, C6, or Huh7 cells rapidly in a 37 °C water bath and transfer them into a T25 or T75 flask containing prewarmed Complete DMEM. Incubate at 37 °C in a humidified environment with 5% CO_2_.
2. Feeding schedule: Spent medium to replace every 2 days. If early acidification or a yellow color shift (pH drop) is observed, you have to change the medium immediately.
3. Monitoring growth: Must follow cell morphology daily under a microscope to maintain continuous logarithmic growth. Cultures should not be allowed to exceed 90% confluency to safeguard cell health and preserve rapid doubling times [20, 24].
4. Harvesting & passaging:
  ○ Must aspirate the spent medium completely.
  ○ Must wash the cell sheet with 1 mL (for T25) or 2 mL (for T75) of room‐temperature 1× PBS, then aspirate the wash.
  ○ 0.25% Trypsin–EDTA (0.5 mL for T25; 1.0 mL for T75) to be added and incubated at 37 °C for 30–60 s, while the flask is tapped gently until cells detach completely.
  ○ To immediately neutralize enzymatic activity, add 2–3 volumes (1.5–3.0 mL) of complete DMEM with FCS.
  ○ Must gently pipette up and down against the flask wall to create a single‐cell suspension.
5. Pre‐Assay Synchronization: Exactly 24 h prior to starting the isolation assay, split and seed the cells onto downstream plates/flasks at a density calibrated to reach 80–85% confluency on the morning of the extraction.

#### Part 2: Harvest & Precision Cell Lysis

CRITICAL BENCH NOTE: Must keep all containers, tubes, buffers, and devices on ice throughout this entire phase and have to work quickly to minimize endogenous enzymatic degradation.

1. Cell scraping: Culture dishes are to be removed from the incubator. Media is to be aspirated, and ice‐cold 1× PBS is to be added. Cells from the plastic surface are gently scraped with a prechilled cell scraper.
2. Serum removal washes:
  ○ Cell suspension to be transferred to a cold conical tube and centrifuged at 600 ***g*** for 5 min at 4 °C.
  ○ Must aspirate the supernatant and cell pellet, then suspend the pellet in 5–10 mL of ice‐cold 1× PBS to remove residual serum proteins.
  ○ The sample should be centrifuged again at 600 ***g*** for 5 min at 4 °C, and this wash step should be repeated a total of two times.
3. Resuspension: Resuspend the final cell pellet in working 1× Homogenization Buffer at a precise ratio of 1 mL of buffer per 8 × 10^6^ cells.
4. Dounce homogenization:
  ○ Transfer the cold suspension into the prechilled 2‐mL tight‐fitting glass Dounce homogenizer.
  ○ Must perform steady, vertical strokes while keeping the apparatus nestled in ice:
    - HEK293 Cells: must do 25 strokes.
    - C6 Cells: must execute 50 strokes.
  ○ Note: Avoid rapid, jerky movements to prevent air bubbles and excessive mechanical shear, which can rupture the membranes of vulnerable organelles.

#### Part 3: Density‐gradient separation of organelles via ultracentrifugation

*Summary*: [1000 ***g*** Pelleting] → [Supernatant Over OptiPrep Gradient] → [1st Ultracentrifuge: 5 h] → [Fraction Screen] → [2nd Ultracentrifuge: 2 h Wash]

**Fig. 2:**
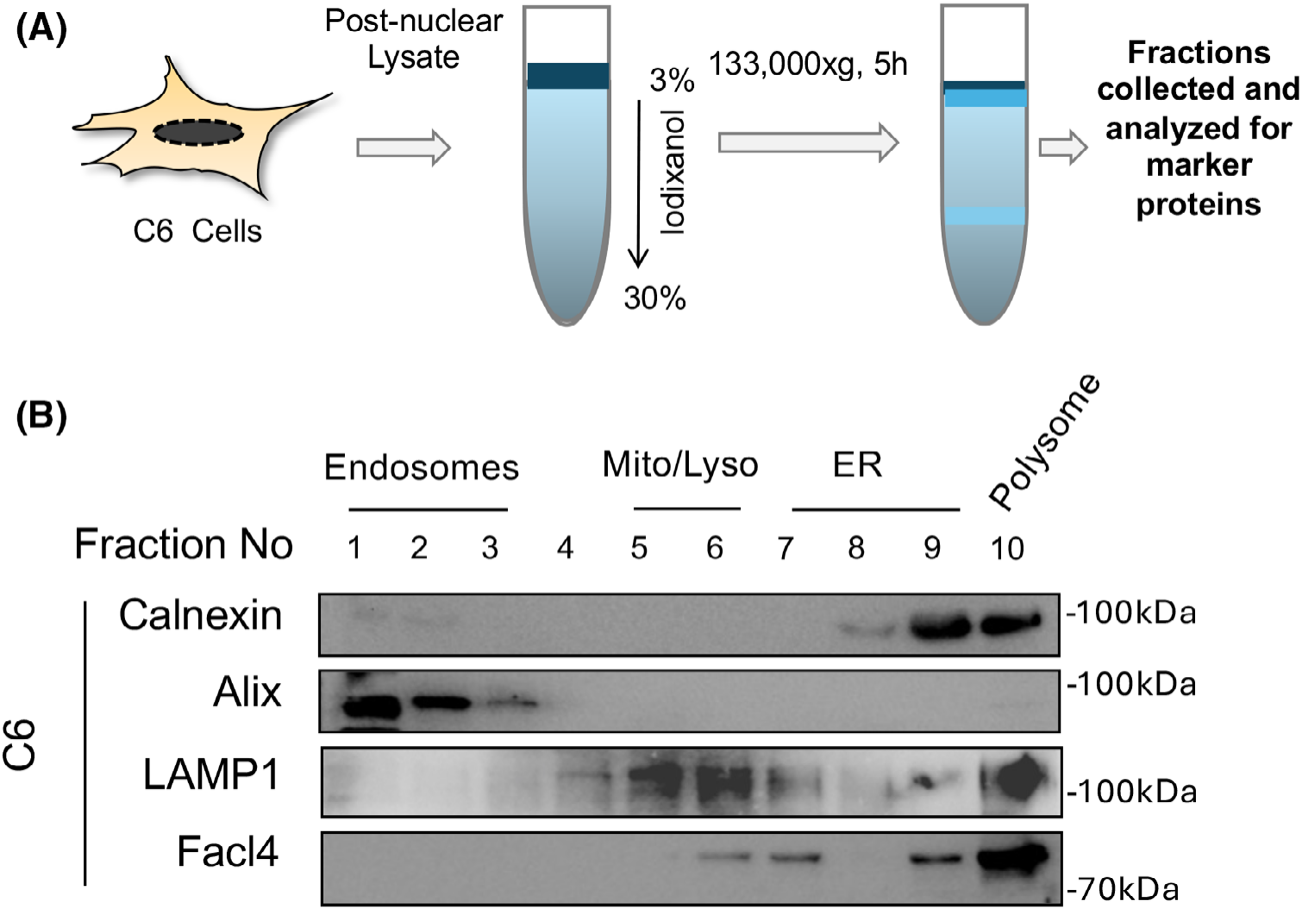
Separation of endosomes and other subcellular organelles by iodixanol density‐gradient centrifugation. (A) Schematic workflow of subcellular fractionation of C6 cell lysate on 3–30% continuous iodixanol density gradient by ultracentrifugation and collection of fractions. (B) Western blots of various organelle‐specific marker proteins to detect in the fractions obtained from subcellular fractionation of C6 postnuclear lysate separated on 3–30% iodixanol gradient. The lysosomal marker LAMP1 and mitochondrial marker Facl4 are absent in ALIX‐positive (endosome) enriched fractions. Figure reproduced from Ref. 26 (Ghosh et al., 2024) [26].

**Fig. 3:**
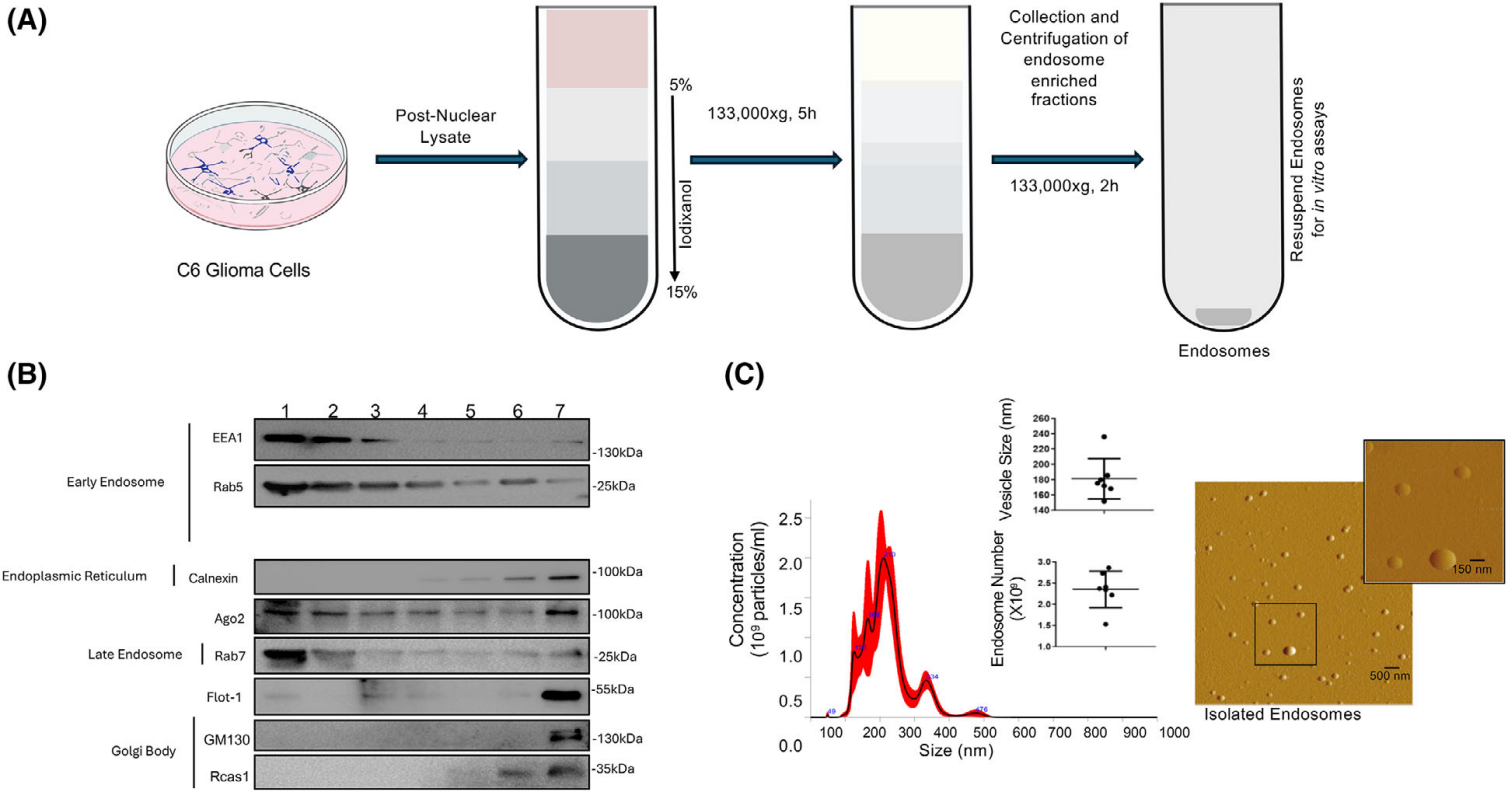
Isolation of the endosomes from C6 glioblastoma cells and characterization of the endosomes. (A) The scheme for isolating endosomes from cell lysates using OptiPrep density‐gradient centrifugation in C6 glioblastoma cells. (B) Immunoblot analysis of the fractions collected from iodixanol optiprep gradient as described in panel A, for different marker proteins of early and late endosomes, ER, and mitochondria. The top fractions 1–3 are collected for the re‐isolation of the endosomes, as depicted in panel A, to get pure endosomes for the RNA import assay. (C) Nanoparticle tracking analysis of the isolated endosomes was done to get a size distribution profile. Average number and size, determined by nanoparticle tracking analysis (NTA) analysis (from triplicate samples), were plotted in the inset panels. The atomic force microscope images of the endosomes and a zoomed inset showing the size and spherical shape of the isolated endosomes (*n* = 3). Error bars represent mean ± SD. The scale bar in panel C represents 500 nm (original) and 150 nm (zoomed inset). Panel C is reproduced from Ref. 26 (Ghosh et al., 2024) [26]. The Sketch‐to‐Figure Feature of the FigureLabs online software was used to generate the cartoon of Panel A.

1. Nuclear cleanout: Transfer the crude cell lysate into a cold microcentrifuge tube. Centrifuge at 1000 ***g*** for 10 min at 4 °C. These steps remove nuclei and unbroken cells. Carefully pipette the clear, organelle‐containing supernatant into a clean tube on ice, and discard the pellet.
2. Pouring the discontinuous Gradient: Use a high‐precision pipette or a long‐needle syringe to construct the gradient interfaces inside a clear ultracentrifuge tube:
  ○ Need to place 1 mL of 15% iodixanol solution at the bottom of the tube.
  ○ Layer 1 mL of 10% iodixanol directly on top of the 15% layer by running it down the interior tube wall slowly.
  ○ Then 1 mL of 5% iodixanol should be placed over the 10% layer. Have to ensure sharp, visible boundary interfaces remain between the liquid densities.
3. Sample loading: The 1000 ***g*** supernatant should be layered directly on top of the 5% iodixanol layer.
4. First ultracentrifugation (Isopycnic Separation): Ensure that the tubes are precisely within 0.01 g weight difference and are loaded into the precooled swing‐bucket rotor. Have to spin at 133 000 ***g*** for 5 h at 4 °C, with the brake set to a slow deceleration profile to protect gradient interfaces.
5. Fraction harvesting: Exact 7 equal‐volume fractions are to be sequentially collected starting from the top layer down to the bottom of the tube, and each fraction is to be placed into a dedicated tube on ice.
6. Screening & pooling: Analyze an aliquot of each fraction via western blot (see Phase 4). Look for enriched bands of early endosome markers (EEA1, Rab5). Pool fractions 1, 2, and 3 (the top layers), which typically contain the purified endosomal populations.
7. Organelle washing & concentration:
  ○ Dilute the pooled endosomal fraction 1–4 with ice‐cold Endosome Wash Buffer.
  ○ Transfer to a clean ultracentrifuge tube, balance perfectly, and spin at 133 000 ***g*** for 2 h at 4 °C to pellet the endosomes.
8. Resuspension & storage: Carefully aspirate and discard the iodixanol‐containing supernatant. Resuspend the visible endosomal pellet in the original homogenization buffer (without sucrose if downstream assays dictate).
  ○ *Yield estimation*: A standard 90‐mm culture dish typically yields 2–3 × 10^9^ endosomes·mL^−1^.
  ○ *Stability*: Store the sample at 4 °C. Utilize within 24 h for functional assays or cargo loading. Discard after 48 h due to structural membrane breakdown.
  ○ The iodixanol gradient analysis, performed over a 3–30% range, can be used to assess the positions of organelles, such as lysosomes, mitochondria, ER, and polysomes across different fractions, thereby enabling separation from the endosomal fraction. Narrowing the gradient to 5–15% allows for clearer separation between early and late endosomes. Moreover, heavier mitochondrial and ER fractions are not present in the one to three fractions used for downstream analysis, thereby ensuring they are free of lysosomal, Golgi, ER, or mitochondrial fractions [25, 26]. The parts of the method and validation are summarized in Figs 1, 2A,B and 3A,B. Troubleshooting is listed in Table 1.

**Table 1:** Bench troubleshooting guide with endosome isolation.

Symptom/failure mode	Probable root cause	Corrective lab action
Low endosome marker yield (EEA1/Rab5 absent in fractions)	Incomplete or inefficient mechanical cell lysis.	Increase the Dounce homogenizer count by 5–10 strokes. Verify that you are using a Type A ‘tight’ clearance pestle.
Ruptured organelles/High cytosolic protein contamination	Over‐homogenization or warming up of samples during processing.	Keep the Dounce glass housing embedded in an ice‐water slurry. Do not exceed the designated stroke limits (25 for HEK, 50 for C6).
Smeared/Blurred gradient interfaces	Fast deceleration profiles or rough handling of tubes post run.	Turn off the centrifuge brake entirely or program an ultra‐slow deceleration ramp profile. Handle tubes vertically with care.
Large salt crystal artifacts covering AFM images	Residual mineral buffer salts from the storage buffer.	Perform a brief, gentle wash step of the dried mica spot using 5 μL of autoclaved ultrapure water, then dry immediately before imaging.

#### Part 4: Downstream characterization modalities of endosomes

##### Method A: Atomic force microscopy

1. Sample preparation: Take an aliquot of the concentrated endosomal pellet and resuspend it thoroughly in autoclaved ultrapure water to minimize salt crystallization during drying.
2. Deposition: Pipette 5 μL of the diluted endosomal suspension onto a freshly cleaved mica sheet (Muscovite Mica‐V1).
3. Drying protocols: Allow the drop to air‐dry flat for 15 min at room temperature. If salt artifacts or interfering molecules are anticipated, place a 10 μL droplet of autoclaved ultrapure water onto the spot for 5 s, tilt to rinse, immediately wick away excess liquid with filter paper, and allow to dry completely.
4. Instrument operation: Install a microfabricated silicon cantilever (225 μm length). Mount the mica sheet into a PicoPlus 5500 ILM AFM system. Execute imaging exclusively in AC (Tapping) Mode, utilizing a piezo scanner calibrated to a maximum 9 μm lateral travel range. Process raw topographic files using Picoview 1.1 software (Fig. 3C).

##### Method B: Nanoparticle tracking analysis (NTA)

1. Fluid path fluidics: Flush the Nanosight NS300 system with 2 mL of sterile 1× PBS to ensure the background particle count is <1 particle/frame.
2. Sample dilution:
  ○ *EVs*: Resuspend the EV pellet in 1× PBS and make a 10‐fold dilution (e.g., 100 μL sample + 900 μL PBS) to a total volume of 1 mL.
  ○ *Purified Endosomes*: Resuspend the endosomal pellet directly in 1 mL of 1× PBS without secondary serial dilutions.
3. Injection & acquisition: Load the sample into a sterile syringe. Inject into the Nanosight NS300 laser sample chamber until the fluid path is completely primed without air bubbles. Capture three separate 60‐s video tracks under temperature‐controlled settings (25 °C). Analyze particle hydrodynamic diameters using the tracking wizard (Fig. 3C).

##### Method C: Marker verification via immunoblotting

1. Denaturation & electrophoresis: Combine your protein lysate/fraction specimens with SDS/PAGE loading buffer containing reducing agent. Heat at 95 °C for 5 min. Resolve samples using standard SDS/PAGE mini‐gels.
2. Electrotransfer: Transfer proteins from the polyacrylamide gel matrix onto a PVDF nylon membrane via wet tank transfer setup at 4 °C overnight.
3. Blocking: Submerge the membrane in a 3% bovine serum albumin (BSA) blocking solution made in 1× TBS‐Tween for exactly 1 h at room temperature under gentle agitation.
4. Primary antibody incubation: Incubate membranes with validated specific primary antibodies (e.g., anti‐EEA1 or anti‐Rab5) diluted in blocking buffer for a minimum of 16 h at 4 °C.
5. Secondary conjugate incubation: Wash membranes three times with TBS‐Tween. Incubate for 1 h at room temperature with horseradish peroxidase (HRP)‐conjugated secondary antibodies diluted at 1:8000.
6. Chemiluminescent development: Wash the membrane three times. Apply ECL substrate and capture digital blot exposures via a UVP BioImager 600 system running VisionWorks Life Science software (version 6.8).

##### Protocol summary

- Purity check: Verify the complete absence of mitochondrial and ER contaminants.
- Fraction selection: Collect only the second, third, and fourth gradient fractions to avoid other organelle‐derived microRNAs.
- Vesicle integrity: Confirm the intact, spherical structure of the endosomes before starting any *in vitro* assays.
- Analytical tools: Use NTA and AFM for quality control.

##### Note for western blot verification

To ensure your isolated endosomes (Fractions 1–3 in 5–15% or 1–4 in 3–30% gradients) are strictly free from microRNA‐rich mitochondrial and ER contamination, a comparative western blot alongside positive markers to be tested (Figs 2A,B and 3A,B).

##### Sample preparation

- Target concentration: Separate 10–20 μg of total protein per lane
- Controls: Always load total cell lysate (TCL) as a positive control for all cellular organelles. The purity of the endosomes, particularly the absence of mitochondrial and ER contamination—two primary sources of microRNAs (miRNAs)—must be confirmed. Fractions devoid of markers from these organelles, specifically the second to fourth fractions of the gradient (as shown in Figs 3A,B and 2A,B) [26], were collected to re‐isolate endosomes free from mitochondrial and ER contamination. The integrity and spherical morphology of the isolated vesicles were verified by NTA and AFM on the isolated endosomes prior to their use in *in vitro* assays (Fig. 2C) [26]. Antibody details are in Table 2.

**Table 2:** Primary antibody panel.

Subcellular target	Selected western blot markers	Status in Fraction 1–3	Purpose
Endosomes (Target)	EEA1 (early endosomes), Rab7 (late endosomes)	Strong enriched band	Confirms target recovery
Endoplasmic reticulum	Calnexin, GRP78/BiP	No band visible	Confirms absence of ER
Mitochondria	COXIV, TOMM20	No band visible	Confirms absence of mitochondria

### RNA substrate preparation for endosome import assay

#### Part 5: Reagent Preparation & Setup

- Thaw on ice: Thaw synthetic miR‐122 RNA (22 nt), T4 PNK enzyme, ATP, and 10× T4 PNK buffer.
- Prechill centrifuge: Set your microcentrifuge to 4 °C for the downstream TRIzol/chloroform phase separation.
- Sanitize workspace: Wipe down pipettes and the benchtop with an RNase decontamination solution (e.g., RNase Away).

#### Part 6: 5′ end‐labeling reaction

- Assemble reaction: In a sterile, nuclease‐free tube on ice, combine:
  ○ 50 pmol synthetic miR‐122 RNA
  ○ 10× T4 PNK buffer (to a final 1× concentration)
  ○ ATP (to a final 1 mm concentration)
  ○ T4 PNK enzyme (10 U/μL, use the manufacturer‐recommended volume)
- Mix & spin: Mix the reagents gently by flicking, then briefly spin in a microcentrifuge to collect the liquid at the bottom.
- Incubate: Place the tube in a water bath or thermal block at 37 °C for 30 min. Do not agitate.
- Stop reaction: Add an appropriate volume of Tris‐EDTA (TE) buffer to the tube and mix gently.

#### Part 7: Column Purification & Phase Separation

- Spin column: Apply the complete reaction mixture to a Roche Mini Quick Spin Oligo Column.
- Collect RNA: Centrifuge the column according to the manufacturer's speed and time specifications. Collect the flow‐through.
- Add TRIzol: Add TRIzol LS directly to the collected flow‐through sample.
- Add chloroform: Add chloroform to the mixture.
- Separate phases: Mix the tube thoroughly by inversion or vortexing. Centrifuge at maximum speed at 4 °C to separate the layers.
- Transfer aqueous phase: Carefully pipet the top, clear aqueous phase into a clean, sterile tube. Avoid touching the interphase.

#### Part 8: Precipitation overnight

- Add co‐precipitant: Add GlycoBlue co‐precipitant to the transferred aqueous phase.
- Add alcohol: Add isopropanol to the tube and mix thoroughly by inversion.
- Precipitate: Incubate the mixture at −20 °C overnight to precipitate the labeled RNA [26].

#### Part 9: RNA Recovery & Quantitation

- Pellet RNA: Centrifuge the sample at maximum speed at 4 °C for at least 15–30 min to form a blue pellet.
- Remove supernatant: Carefully aspirate and discard the liquid supernatant without disturbing the blue RNA pellet.
- Wash pellet: Add 70% ethanol to the tube and centrifuge again for 5 min.
- Air‐dry: Carefully remove the ethanol supernatant and let the pellet air‐dry briefly (do not over‐dry).
- Resuspend: Dissolve the pellet in nuclease‐free water.
- Dilute: Adjust the final volume with nuclease‐free water to reach a final concentration of 1 pmol/μL (1 μm).
- Store: Aliquot and store the labeled RNA at −20 °C or −80 °C.

For Notes and Troubleshooting consult Table 3.

**Table 3:** Possible problems with RNA substrate preparation.

Problem/symptom	Possible cause	Troubleshooting step/solution
No visible blue pellet	Incomplete precipitation	Incubate at −20 °C for a full 12–16 h, or move to −80 °C for 2 h.
	Centrifugation too slow or short	Spin at maximum speed 12 000 ***g*** at 4 °C for at least 30 min.
	Lost during ethanol wash	Always orient the tube hinge outward so you know exactly where the pellet sits when aspirating.
Low RNA concentration	Pellet did not fully dissolve	Do not over‐dry the pellet. Redissolve it by gently pipetting or shaking at room temperature for 10 min.
	Incomplete aqueous phase collection	Maximize phase separation by spinning for 15 min. Leave a sliver of the aqueous layer to avoid interphase.
Incomplete 5′ end‐labeling	5′ position already blocked	Ensure your synthetic RNA supplier did not add a pre‐existing 5′ phosphate. If they did, treat with CIP phosphatase first.
	Degraded ATP or PNK enzyme	Avoid repeated freeze–thaw cycles. Aliquot buffers and enzymes into single‐use portions.
	RNase contamination	Use fresh nuclease‐free water. Clean your workspace, racks, and pipettes with an RNase decontamination spray.
Poor phase separation	Incorrect reagent ratio	Check that your volume ratio of TRIzol LS to chloroform strictly matches the manufacturer's specification.
	Centrifugation temperature too high	Perform the TRIzol and chloroform centrifugation strictly at 4 °C to keep the phases stable.
Column failure/low recovery	Column overloaded or bypassed	Check the Roche Mini Quick Spin Oligo Column manual to ensure your sample volume does not exceed limits.

### Endosomal import assay with synthetic RNA substrate

#### Core Experimental Design & Controls

- Experimental replicates: Perform all loading reactions and qPCR assays in triplicate (*n* = 3) to allow for valid statistical analysis.
- Internal normalization control: For these *in vitro* assay samples, use endogenous endosomal miR‐146a to normalize the levels of your synthetic target miRNA (e.g., synthetic miR‐122). Do not use U6 snRNA, as U6 is reserved for cellular samples.
- Critical handling rule: Keep all buffers, reagents, and vesicle samples on ice at all times unless a specific incubation temperature is stated. Handle all steps gently to avoid mechanical shearing of the vesicles.

#### Part 10: Reagent & Mixture Preparation

- Stock target miRNA: Ensure your synthetic target miRNA is 5′‐phosphorylated. Resuspend or dilute it to a stock concentration of 1 μm using strictly RNase‐free water.
- Endosome quantification: Ensure your isolated endosome stock is characterized and quantified so that you can reliably aliquot 2 × 10^9^ endosomes per 50‐μL reaction. Verify purity beforehand using endosome‐specific markers.

#### Part 11: Cargo‐loading reaction

**Fig. 4:**
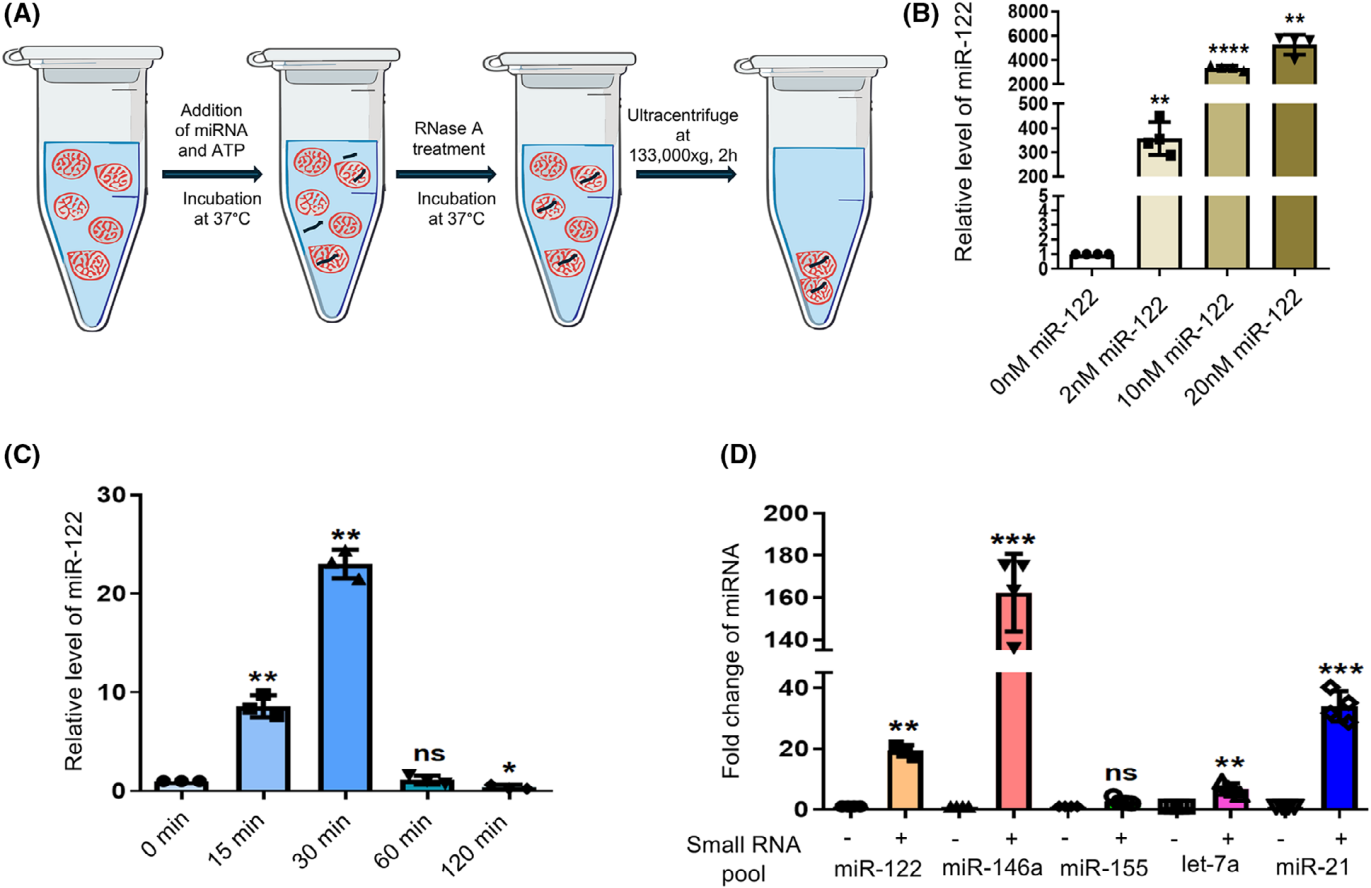
*In vitro* RNA import assay with C6 glioblastoma cells isolated endosomes. (A) A schematic presentation of the strategy of the import assay, followed by RNase treatment to remove non‐imported microRNA (miRNA) substrate, and recovery of endosomes with imported miRNA protected from RNase. (B) Amount of miR‐122 imported and protected from RNase with increasing substrate concentration. The imported miRNA was quantified by quantitative reverse transcription polymerase chain reaction (qRT‐PCR). Endogenous miR‐146 level present in endosomes was used for normalization (*n* = 4 independent experiments). (C) Relative amount of synthetic miR‐122 (10 nm) as cargo that gets imported with increasing time of incubation in an import buffer with isolated endosomes followed by an RNase treatment to get rid of the nonimported miRNAs. The imported miRNA was quantified by qRT‐PCR and the value at 0 min of incubation was considered as unit. Endogenous miR‐146 level present in endosomes was used for normalization (*n* = 3 independent experiments). (D) Differential packaging of miRNAs into endosomes from a small RNA pool. Small RNA pool from C6 cells was incubated with isolated endosomes and the level of imported and RNase‐protected levels of different miRNAs were measured. The imported miRNA was quantified by qRT‐PCR and in each case, the value without small RNA pool added in the incubation was considered as unit. Endogenous miR‐146a level present in endosomes was used for normalization (*n* ≥ 3 independent experiments). Data information: In all the experimental data, error bars are represented as mean with SD, ns, nonsignificant, *P < 0.05, **P < 0.01, ***P < 0.001, ****P < 0.0001, respectively. *P*‐values were calculated by a two‐tailed paired t‐test in most of the experiments unless mentioned otherwise. The relative level of miR‐122 was normalized with the miR‐146a level by the 2^−ΔΔCt^ method. Fold change of miRNA was calculated by the 2^−ΔCt^ method. Panels B, C, and D were reproduced from Ref. 26 (Ghosh et al., 2024) [26]. The Sketch‐to‐Figure Feature of the FigureLab online software was used to generate the cartoon in panel A.

**Fig. 5:**
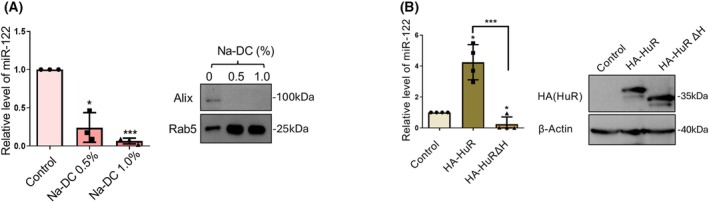
Parameter variability affects endosomal import efficacy of substrate RNA. (A). Imported microRNAs (miRNAs) are protected from RNase. After the import reaction, an increasing concentration of ionic detergent sodium deoxycholate was added to rupture the endosomes and expose the imported miR‐122 to RNase A (100 ng·μL^−1^) for degradation (upper panel). The level of miRNA, with or without detergent treatment of the endosome, was quantified by quantitative reverse transcription polymerase chain reaction (qRT‐PCR). Endogenous miR‐146a present within endosomes was used for normalization (*n* = 3 independent experiments). Immunoblot of RNase and detergent‐treated vesicles showing the amount of endosomal protein Rab5 that was not solubilized by the detergent from the endosomal membrane, while Alix was extracted with the detergent applied. (B) Effect of deletion of the hinge region of HuR, necessary for its ubiquitination and miRNA unbinding, on endosomal miRNA import [19]. Full‐length HuR (HA‐HuR) and its mutant version with a deletion in the hinge region (HA‐HuRΔH) were expressed in C6 cells, and endosomes were isolated. *In vitro* miRNA import reaction was performed using synthetic miR‐122 as substrate, and the internalized miR‐122 level was measured by qRT‐PCR, and relative levels of imported and RNase‐protected RNA were plotted. The level of miR‐122 imported in the control non HA‐HuR expressing cell‐derived endosome set was considered as a unit (*n* = 4 independent experiments). Immunoblots indicate the expression level of HA‐HuR and HA‐HuRΔH in C6 cells used for endosome isolation. Data information: In all the experimental data, error bars are represented as mean with SD, ns, nonsignificant, *P < 0.05, ***P < 0.001, respectively. *P*‐values were calculated by a two‐tailed paired t‐test in most of the experiments unless mentioned otherwise. The relative level of miR‐122 was normalized with the miR‐146a level by the 2^−ΔΔCt^ method. Fold change of miRNA was calculated by the 2^−ΔCt^ method. Molecular weight marker positions are indicated and shown on the respective western blots. Reproduced from Ref. 26 (Ghosh et al., 2024) [26].

**Fig. 6:**
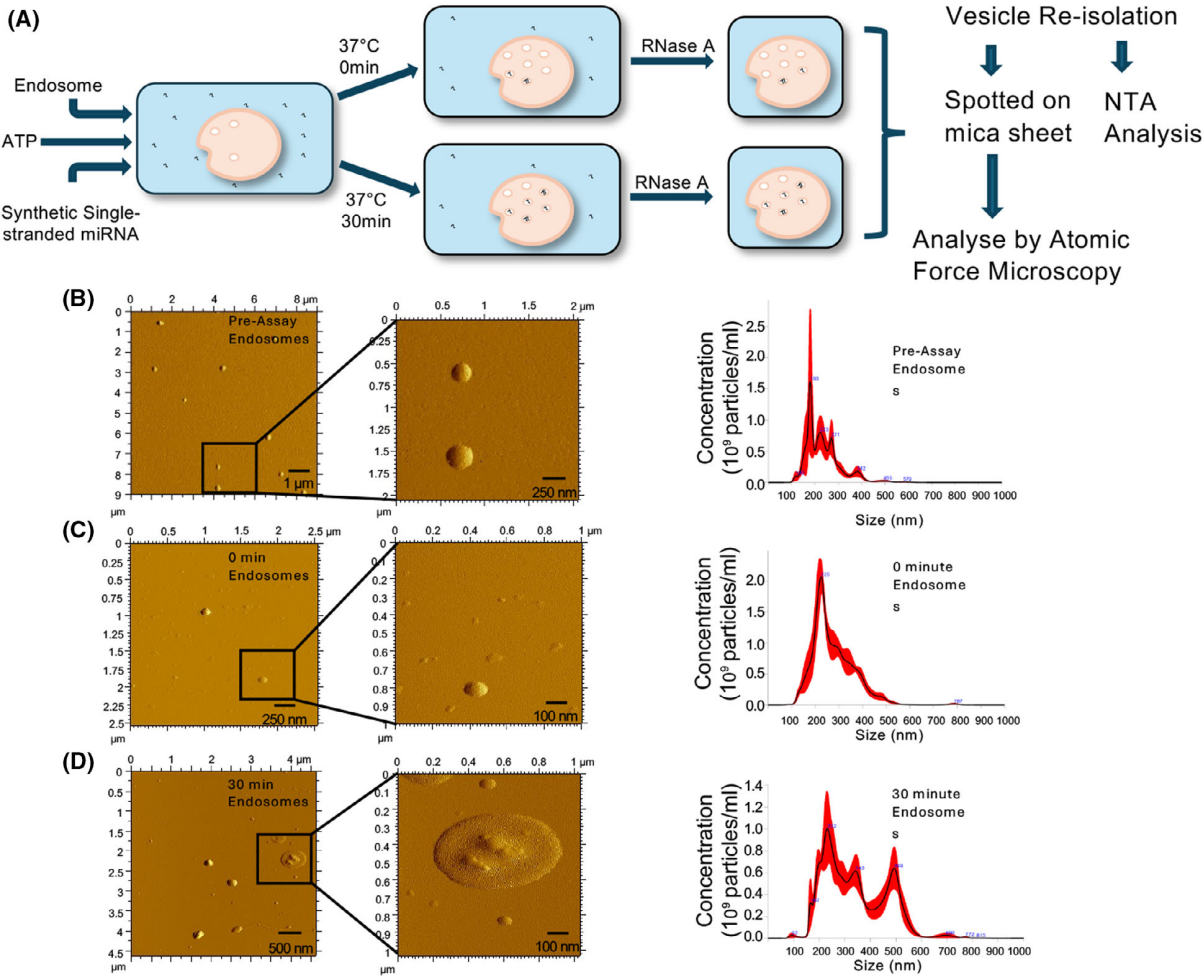
Maturation of endosomes to larger Multivesicular Structures *in vitro*. (A) Schematic diagram of the study of *in vitro* maturation of isolated endosomes. (B–D) Atomic force micrograph and nanoparticle tracking analysis (NTA) analysis of isolated endosomes prior to cell‐free assays (B); or after 0 min (C) or after 30 min (D) of incubation. Insets show zoomed, higher‐resolution images. Analysis of their concentration and size by NTA. Scale bars for Panel B are 1 μm and 250 nm (zoomed part); for Panel C are 250 nm and 100 nm (zoomed part); for Panel D are 500 nm and 100 nm (zoomed part). Reproduced from Ref. 26 (Ghosh et al., 2024) [26].

1. Assemble the reaction: In a 1.5‐mL RNase‐free microcentrifuge tube, set up a 50‐μL total volume reaction containing:
  ○ 2 × 10^9^ isolated endosomes.
  ○ Optimized synthetic miRNA at a final concentration between 1 nm and 30 nm.
  ○ RNase‐free buffer to bring the total volume to 50 μL.
2. Incubate: Place the tubes into a thermomixer equipped with a 1.5‐mL thermoblock.
3. Settings: Set the temperature to 37 °C with gentle agitation.
4. Duration: Incubate for exactly 30 min.
  ○ *Note*: While the protocol permits a 0–120 min range, 30 min is the optimal threshold. Exceeding this time compromises vesicle integrity and decreases packaging efficiency (Fig. 4A,C)
  ○ Use of the cellular small RNA pool (100 ng/50ul reaction) used for import shows the import specificity/preference for RNA substrate, and use of a synthesis substrate (single‐stranded synthetic miR‐122) at a concentration range showed dose‐dependent increase of import level (Fig. 4A–D).
  ○ To confirm the import of miRNA into the lumen of endosomes, rather than mere association on their surface, an RNase treatment in the presence of deoxycholate is needed to detect the increase in RNAse sensitivity with Detergent treatment (Fig. 5A). However, including scrambled RNA as a negative control would be an important choice to rule out noise from adsorbed RNA on the endosomal surface.
  ○ AFM of the reisolated endosomes can also confirm the integrity of the reisolated endosomes after the reaction.
  ○ Use of endosomes from different cells expressing miRNA binder and export regulatory protein HA‐HuR or its truncated version that cannot bind miRNA showed a difference in miRNA import levels, confirming that the import process is selective and its efficiency is affected by endosome composition or cell types (Fig. 5B) [26, 27].
  ○ AFM and NTA data suggest vesicle enlargement during the incubation window, which may be consistent with endosomal maturation, though confirmation by electron microscopy and biochemical markers would be required to establish this definitively (Fig. 6) [26, 27].
  ○ The published literature confirms the effects of amyloid proteins [28] or Rab5 [26] on miRNA import levels. It was also noted how one miRNA substrate can affect the import of another substrate miRNA in the *in vitro* reaction. This RNA cooperativity in the import process has been recently explored, and the dependence of cooperative import of HuR protein has been explored [26 ,27].

#### Part 12: RNase Protection assay

This step degrades nonimported, external RNA to eliminate false positives.

1. Add enzyme: Directly add RNase A to the 50‐μL reaction to achieve a final concentration of 0.5 U·μL^−1^.
2. Digest: Incubate the mixture at 37 °C for 5 min with continuing gentle agitation.

#### Part 13: Endosome Re‐Isolation & Washing

1. Pellet vesicles: Transfer the reaction mixture to appropriate ultracentrifuge tubes. Spin at 133 000 ***g*** for 2 h to pellet the loaded endosomes.
2. Wash 1: Carefully aspirate the supernatant containing the digested RNA fragments and RNase A. Resuspend the pellet gently in RNase‐free wash buffer.
3. Pellet Wash 1: Repeat the ultracentrifugation at 133 000 ***g*** for 2 h.
4. Pellet Wash 2: Aspirate the supernatant, gently resuspend the pellet a second time in RNase‐free wash buffer, and repeat the 2‐h spin at 133 000 ***g*** (suggested if residual RNase causing a problem with recovery of imported RNA from pellet).
5. Final pelleting: Thoroughly remove the final supernatant to eliminate all residual free RNA.

#### Part 14: Total RNA extraction

1. Lysis: Lyse the washed endosome pellet by adding TRIzol LS reagent (or a certified RNase‐free RNA Isolation Kit) directly to the pellet, following the manufacturer's protocol
2. Co‐precipitation: Because endosomal miRNA yields are typically low, add glyco‐blue co‐precipitant directly to the sample mixture.
3. Precipitation: Precipitate the total RNA by adding isopropanol according to standard TRIzol processing steps. Centrifuge to pellet the RNA, wash with ethanol, air dry briefly, and dissolve in RNase‐free water.

#### Part 15: Two‐step RT‐qPCR quantification

##### Step A: Complementary DNA (cDNA) synthesis

1. Aliquoting: Take an equivalent volume of the purified endosomal RNA isolated from your *in vitro* assay.
2. Reaction: Combine the RNA with a specialized miRNA reverse transcription kit (Applied Biosystems) and target‐specific RT primers.
3. Synthesis: Run the reverse transcription reaction according to the kit instructions to generate stable cDNA.

##### Step B: Real‐time PCR amplification

1. Master mix formulation: Prepare a PCR master mix for your replicates using:
  ○ TaqMan Universal PCR Master Mix No AmpErase.
  ○ Specific TaqMan reagents (primers and fluorescent probes) dedicated to your target miRNA and your normalizer (miR‐146a).
2. Reaction loading: For each PCR well, utilize exactly one‐third of the total reverse transcription mixture volume as your cDNA template.
3. Execution: Plate all samples in triplicate onto a Bio‐Rad CFX96™ real‐time system. Run the standard TaqMan thermocycling protocol.

#### Part 16: Data & Statistical Analysis

1. Ct Extraction: Export the threshold cycle values from the Bio‐Rad software.
2. Normalization: Calculate the relative expression of your synthetic target miRNA using the Delta CT method, utilizing the endosomal miR‐146a Ct values as your internal housekeeping control.
3. Software Input: Import your final normalized triplicate values into GraphPad Prism 5.00.
4. Hypothesis testing: Run a Student's *t*‐test to compare experimental groups (e.g., different loading concentrations or time points).
5. Significance threshold: Define statistical significance at *P* < 0.05.
6. Graphing: Plot your data with error bars explicitly representing the mean ± standard deviation.

## Results and Discussion

The assay developed using endosomes isolated from C6 glioblastoma cells should be reproducible across other cell types, as discussed in the original publications [26]. This assay does not require the addition of a cytosolic concentrate to import microRNAs (miRNAs). However, the inclusion of specific factors, such as recombinant HuR, has been shown to stimulate import and, subsequently, enhance the cooperative import of miRNAs, as discussed in our recent report on cooperative miRNA import into endosomes in the presence of catalytic miRNAs [26, 27]. This assay system will serve as an ideal platform for monitoring various steps in the miRNA import process, including the binding of the HuR‐miRNA complex to RalA, the transfer of miRNAs to STX5, and the internalization of the STX5‐miRNA complex into the lumen of endosomes. The roles of specific ESCRT components, accessory factors, Rab5 GTPases, and mTOR complexes warrant further investigation [24, 26, 29]. Screening for factors that contribute to the import of miRNAs into endosomes can be conducted using endosomes isolated from cells subjected to targeted gene deletion via CRISPR/Cas9 or siRNA‐mediated knockdown. Similarly, this assay system will be an essential tool for screening small‐molecule inhibitor libraries to evaluate their effects on miRNA import. The addition of recombinant proteins, such as HuR, may be extended to include Argonaute proteins and their interacting partners to assess their impact on the endosomal loading of miRNAs [19]. Recent research has underscored the importance of proteins, such as YBX1, La, and heterogeneous nuclear ribonucleoproteins (hnRNPs) in recognizing miRNA export signals, highlighting their role in the miRNA export process [9, 14, 15, 16, 17, 18, 19, 20, 21]. Exploring these factors further, through *in vitro* miRNA export assays, could provide valuable insights into their regulatory roles [2].

Viral RNA and proteins are known cargoes of EVs released by infected cells. This assay system may be employed to investigate how viral RNAs and proteins, such as HIV‐1 Tat and TAR, are packaged within endosomes. Such studies will facilitate the exploration of antiviral factors that influence viral RNA and protein loading into endosomes, thereby affecting the EV cargo profile and possibly the infectivity of HIV‐1 in infected macrophages and T cells [30, 31, 32].

The assay suggests the conversion of early endosomes to late endosomes or MVBs that depends on ATP and Rab5. It is interesting to explore the molecular players that affect the conversion between early and late endosomes. The effect of inhibiting this conversion on loaded miRNAs will be an interesting aspect to test *in vitro*. Overall, this assay is an innovative and reproducible system for monitoring cargo loading inside endosomes and may be useful for exploring the cargo loading and export processes in diverse types of higher eukaryotes [26, 27, 33].

## Tips and tricks

Here is a concise list of practical tips, tricks, and critical warnings compiled from the protocol to maximize your assay's success and prevent common points of failure:

### Cell Culture & Harvesting Optimization

- Prevent overcrowding: Never let cells exceed 90% confluency. Overcrowding compromises cell health, shifts morphology, and slows down doubling time.
- Control scale‐Up: When scaling up for larger assays, always split cells into flasks with exactly twice the surface area to maintain predictable growth rates.
- Remove serum contamination: Wash your harvested cell pellet thoroughly twice with ice‐cold PBS. Residual serum proteins can easily contaminate downstream western blots.

### Endosome Isolation & Gradient Precision

- Strict temperature control: Keep all tubes, buffers, rotors, and the Dounce homogenizer completely on ice. Ambient temperatures will rapidly degrade or prematurely rupture the organelles.
- Tailor your lysis: Match your Dounce homogenization strokes precisely to your cell line (25 strokes for HEK293/50 strokes for C6). Over‐homogenization will rupture the endosomal membranes.
- Protect the gradient interfaces: When building your discontinuous OptiPrep gradient (15%, 10%, and 5%), gently pipet each layer down the side of the tube. Sharp, unmixed boundaries between layers are essential for proper separation.
- Target the sweet spot: Focus your downstream collection on Fractions 1, 2, and 3. These fractions are highly enriched for endosomes while remaining free from major miRNA‐contaminating organelles like the ER and mitochondria.

### RNA substrate integrity

- Mandatory 5′ phosphate: Ensure your synthetic miRNA is ordered with a 5′ phosphate modification. This modification is non‐negotiable for proper RISC loading and downstream silencing activity.
- Boost low‐yield recovery: When recovering low amounts of RNA during precipitation, always include GlycoBlue co‐precipitant and allow an overnight incubation at −20 °C to maximize your pellet size.
- Zero agitation: Keep the tube completely still during the T4 PNK 5′ end‐labeling incubation at 37 °C; shaking or agitation can reduce kinase efficiency.

### miRNA Loading & Protection Assays

- Stick to the 30‐minute window: Do not let the cargo‐loading reaction run longer than 30 min. Extended incubation times cause endosomes to lose structural integrity and decrease overall packaging efficiency.
- Eliminate false positives: Never skip the RNase A protection step. Treating the mixture with RNase A ensures that any remaining external, nonimported miRNA is entirely degraded, leaving only truly internalized cargo for quantification.
- Expect morphological changes: Do not be alarmed if structural analysis changes over time. During the 37 °C incubation period, early endosomes will naturally mature into late endosomes and multivesicular bodies.
- After ultracentrifugation, miRNA is either inside pelleted endosomes or free in supernatant, but only the pellet is measured. Quantifying miRNA in the supernatant at 30 and 60 min would distinguish the options. miRNA in supernatant at 60 min indicates vesicle leakage; if negative at both points, it suggests RNase penetration or vesicle loss. These may be of consideration if the assay needs to continue beyond the recommended 30‐min time window.

### Accurate PCR normalization

- Switch your normalizers:
  ○ Use U6 snRNA *only* when quantifying internal cellular control samples.
  ○ Use endogenous miR‐146a when normalizing your *in vitro* imported synthetic miR‐122 samples to ensure accurate mathematical scaling.

## Conflict of interest

The authors declare no conflicts of interest.

## Author contributions

SG participated in the data curation, formal analysis, investigation, and methodology. KM contributed to the data curation, formal analysis, investigation, as well as writing, review, editing, conceptualization, methodology, and supervision. SNB was engaged in data curation, formal analysis, investigation, writing, review, editing, conceptualization, funding acquisition, methodology, and supervision.

## Data Availability

The data used in this manuscript in Figures 2, 3, 4 and 5 were obtained from a previously published study [26] by these authors in the Journal of Biological Chemistry (Elsevier Inc) for which written permission from the Publisher has been obtained. There is no protein or RNA sequencing data used in the manuscript to deposit in the repository domains.
